# Successful rescue of disseminated varicella infection with multiple organ failure in a pediatric living donor liver transplant recipient: a case report and literature review

**DOI:** 10.1186/s12985-015-0311-7

**Published:** 2015-06-17

**Authors:** Naoya Yamada, Yukihiro Sanada, Noriki Okada, Taiichi Wakiya, Yoshiyuki Ihara, Taizen Urahashi, Koichi Mizuta

**Affiliations:** Department of Transplant Surgery, Jichi Medical University, 3311-1 Yakushiji, Shimotsuke-shi, Tochigi 329-0498 Japan

**Keywords:** Disseminated varicella-zoster infection, Living donor liver transplantation, Multiple organ failure, VZV-DNA PCR

## Abstract

A 12-year-old female patient with biliary atresia underwent living donor liver transplantation (LDLT). Twelve months after the LDLT, she developed acute hepatitis (alanine aminotransferase 584 IU/L) and was diagnosed with disseminated varicella-zoster virus (VZV) infection with high level of serum VZV-DNA (1.5 × 10^5^ copies/mL) and generalized vesicular rash. She had received the VZV vaccination when she was 5-years-old and had not been exposed to chicken pox before the LDLT, and her serum was positive for VZV immunoglobulin G at the time of the LDLT. Although she underwent treatment with intravenous acyclovir, intravenous immunoglobulin, and withdrawal of immunosuppressants, her symptoms worsened and were accompanied by disseminated intravascular coagulation, pneumonia, and encephalitis. These complications required treatment in the intensive care unit for 16 days. Five weeks later, her clinical findings improved, although her VZV-DNA levels remained high (8.5 × 10^3^copies/mL). Oral acyclovir was added for 2 weeks, and she was eventually discharged from our hospital on day 86 after admission; she has not experienced a recurrence. In conclusion, although disseminated VZV infection with multiple organ failure after pediatric LDLT is a life-threatening disease, it can be cured via an early diagnosis and intensive treatment.

## Background

Varicella-zoster virus (VZV) is a DNA virus that belongs to the family of herpes viruses, and is the cause of both chicken pox (as a primary infection) and herpes zoster, which is a reactivation infection that is typically observed in adults. Chicken pox is generally a mild disease, VZV infection is known to be more severe in adolescent patients, and although disseminated VZV infection is a rare disease, it is potentially life-threating in patients with immunosuppression after organ transplantation [1–4]. We had previously reported VZV infections after pediatric living donor liver transplantation (LDLT), which was the largest analysis of VZV infections after pediatric LDLT [5–9]. Based on our findings, we concluded that we could prevent the infection from developing into serious illness via our pre-and post-transplant strategies. However, we recently experienced a case of severe disseminated VZV infection with multiple organ failure (MOF) after pediatric LDLT, and successfully rescued the patient. Therefore we report this case of disseminated VZV infection with MOF after pediatric LDLT, and re-consider the pre-and post-transplant strategies for preventing and treating VZV infection.

## Case presentation

A 12-year-old female patient with biliary atresia underwent LDLT using a left lobe graft from her ABO-compatible father. She had received the VZV vaccination when she was 5-years-old and she had not been exposed to chicken pox before the LDLT. Her serum was positive for VZV immunoglobulin G (IgG) (×160; Artus VZV-PCR kit, Qiagen, USA) at the time of the LDLT. The recipients’ cytomegalovirus (CMV) status was seronegative (IgG-negative and IgM-negative) before the LDLT, although the donor’s CMV status was seropositive (IgG-positive [×1,500] and IgM-negative). Her immunosuppression regimen after LDLT consisted of tacrolimus (FK), methylprednisolone (MP), and mycophenolate mofetil (MMF). She did not receive prophylactic therapy for CMV infection, and was evaluated each month for CMV antigenemia (C7-HRP) [10].

**Fig. 1:**
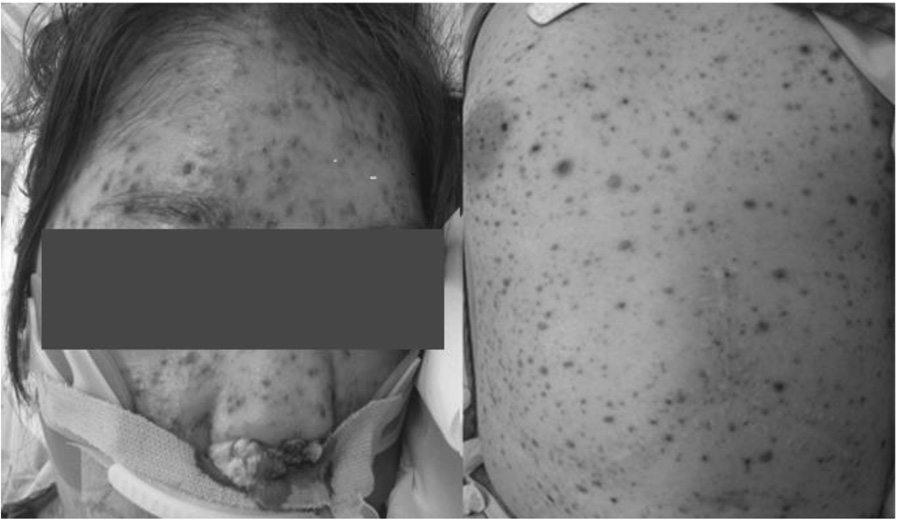
The patient’s skin rash with blisters, which were already partially covered with scabs

Twelve months after the LDLT, she developed a fever and back pain with generalized vesicular rash and was admitted to our hospital 5 days later (Fig. 1). She had not been exposed to any person with chicken pox, she had not been receiving varicella-zoster immune globulin, and she was negative for VZV-IgG antibodies at the hospitalization. We subsequently diagnosed her as having a VZV infection, based on her clinical findings and the PCR results for VZV-DNA (1.6 × 10^5^ copies/mL). As we could not investigate the strain type of the VZV, we were unable to determine whether her infection was primary infection or secondary dissemination. In addition, she exhibited signs of severe hepatic damage (alanine aminotransferase: 584 IU/L) and disseminated intravascular coagulation (DIC) (Table 1). We considered performing a liver biopsy, although we chose not to perform the biopsy, given her bleeding tendency. Based on the findings, we diagnosed her with disseminated VZV infection that was accompanied by acute hepatitis and DIC, and started intensive treatment.

**Table 1:** Laboratory data from admission reveals severe hepatic damaged and disseminated intravascular coagulation

WBC	3,400	/mm^3^	T-bil	3.14	mg/dl
RBC	456×10^4^	/mm^3^	D-Bil	1.54	mg/dl
Hb	14.3	g/dl	BUN	16	mg/ dl
Ht	41.4	%	Cre	0.42	mg/dl
Plt	3.8 ×10^4^	/mm^3^	CRP	0.67	mg/mi
T-P	6.5	g/d	PT-%	44.2	%
Alb	3.5	g/cl	APTT	34.6	sec
CPK	36	IU/L	PT-INR	1.50	
AST	956	IU/L	Fib	168	mg/dl
ALT	584	IU/L	FDP	38.4	μ/ml
ALP	779	IU/L	D-dimer	36.3	μ/ml
LDH	1581	IU/L	EBV-DNA	12x10^2^	copies/1O^6^WBC
r-GTP	583	IUL	CMV-antigen	(−)	

*WBC* white blood cell, *RCB* red blood cell, *Hb* hemoglobin, *Ht* hematocrit, *Plt* platelet count, *T-P* total protain, *Alb* serum albumin, *CPK* cretine phosphokinase, *AST* aspartate transaminase, *ALT* alanine amino transaminase, *ALP* alkaline phosphatase, *LDH* lactate dehydrogenese, *r-GTP* γ-glutamyltranspeptidase, *T-bil* total-bilirubin, *D-Bil* direct bilirubin, *BUN* blood urea nitrogen, *Cre* cretinine, *CRP* c-creative protain, *PT* prothrombin time, *APTT* activated partial trhomboplastin time, *PT-INR* prothrombin time-international normalized ratio, *Fib* fibrinogen, *FDP* fibrin degradetion product, *EBV* Epstein-Barr virus, *CMV* cytomegalovirus

EBV-DNA 12x102 copies/1O6WBC ⇒ EBV-DNA 12x10^2^ copies/10^6^ WBC

**Fig. 2:**
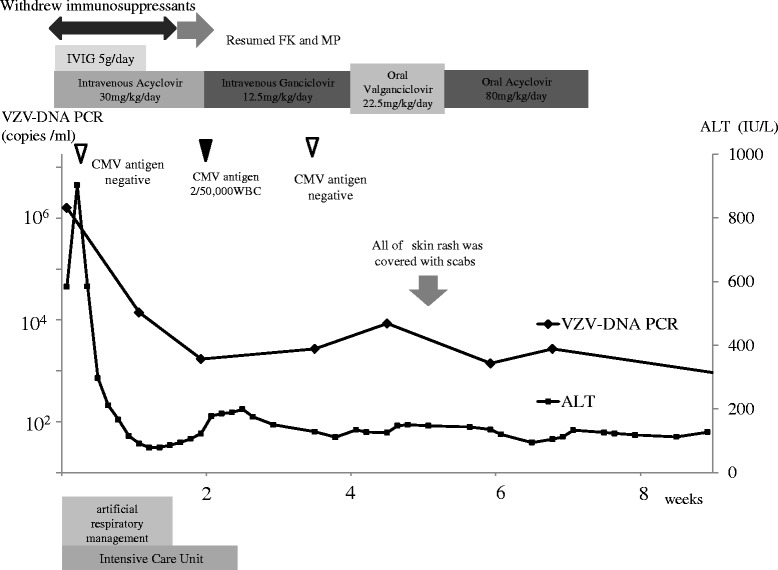
The patient’s clinical course. The patient exhibited severe hepatitis (alanine aminotransferase: 951 IU/mL), which gradually improved. PCR testing for varicella-zoster virus (VZV) DNA revealed decreasing values after treatment with intravenous acyclovir in the first two weeks. However, the VZV-DNA remained high and tended to be elevated after five weeks of antiviral therapy. Therefore we added prophylactic oral acyclovir for two weeks after her clinical symptoms had improved. She was eventually discharged on day 86 after her admission, and has not experienced a recurrence

Although she underwent treatment with intravenous acyclovir (30 mg/kg/day), intravenous immunoglobulin (125 mg/kg/day), and withdrawal of all immunosuppressants (FK, MP, and MMF), her symptoms worsened, and she developed pneumonia that required artificial respiratory management for 11 days (Fig. 2). Based on the clinical course, we assumed that this was VZV pneumonia, although we could not perform a bronchoalveolar lavage to confirm this hypothesis. On day 4 after admission, she exhibited neutropenia (<1000/mm^3^), due to bone marrow suppression. On day 12, she experienced a generalized tonic-clonic seizure. Although brain magnetic resonance imaging did not reveal any abnormal findings, we suspected encephalitis due to disseminated VZV infection and considered performing a lumbar puncture, although we chose not to perform the puncture, based on her unresolved bleeding tendency. Therefore, she remained in the intensive care unit for 16 days, and as her symptoms gradually improved, we resumed the oral immunosuppressants (FK and MP) after 12 days of cessation. In addition, on day 16, we changed the intravenous acyclovir to intravenous ganciclovir (12.5 mg/kg/day), because she had developed a CMV infection (CMV antigen: 2/50,000 white blood cells). After 14 days of intravenous ganciclovir, we changed the treatment to oral valganciclovir after we had confirmed the absence of CMV antigen. At 5 weeks after these treatments were started, all of her skin rash was covered with scabs, her organ symptoms had disappeared, and her clinical findings appeared to be completely improved, although her VZV-DNA levels remained high (8.5 × 10^3^ copies/mL). We continued the oral acyclovir treatment for another 2 weeks, and she was discharged on day 86 after the admission; she has not experienced a recurrence. She was well at 6 months after discharge, and follow-up PCR testing did not reveal any VZV-DNA.

## Discussion

VZV infection is a frequent complication of solid organ transplantation, although most cases are mild and can be treated in the outpatient clinic with oral acyclovir. However, because of their immunocompromised status, some patients develop a severe illness that is known as disseminated VZV infection with visceral involvement, which includes pneumonitis, hepatitis, meningoencephalitis, glomerulonephritis or hemorrhagic complications, and may result in MOF. Fehr et al. have reported that the mortality rate for disseminated VZV infection after renal transplantation is as high as 34 % [1]. Rommelaere et al. have also recently reported that the mortality rate of disseminated VZV infection after renal transplantation appears to have decreased since 1995, although no improvements have been observed over the last 10 years [11]. We have summarized the recently reported cases of disseminated VZV infection that led to MOF in Table 2 [12–23]. Immunocompromised patients, including solid organ or bone marrow transplant recipients, have the potential to experience a disseminated VZV infection with MOF, and it is noteworthy that the mortality rate is high once VZV infection leads to MOF. Early diagnosis and treatment are critical to rescuing these patients, and liver transplantation should be considered in cases with fulminant hepatic failure.

**Table 2:** Recently reported cases of varicella-zoster infection that ledto multiple organfailure

	Author	Age Sex	Patient background	Types of organ symptoms	Main Treatment	Prognosis
Solid organ transplantation associated	Alvite-Canos [12]	43 M	Heart transplanatation	Hepatitis, Encephalopathy	IVIG, Acyclovir Liver Transplantation,	Alive
	verleden [13]	30 M	Lung transplantation	(hepatic, polmunary, renal insufficiency	Acyclovir	Dead
	Our patient	13 F	LDLT	Hepatitis, DIC, Pneumonia Encephalopathy	Acyclovir, IVIG withdrew the immunosuppressants	Alive
	Spring field C [4]	25 M	Crohn disease	pulmonary	Acyclovir	Dead
	Hagiya [14]	75 F	Acute pancreatitis	Pneumonia, Hepatitis	Acyclovir, topical vidarabine	Dead
	Hirose [15]	13 F	SLE ALL	Hepatitis. DIC. cardiac muscle, Pneumonia. Encephalopathy	Acyclovir, IVIG, Plasma exclian2e	Dead
	Kim [16]	8 M	ALL	ARDS, Hepatitis DIC, encephilopathy	Acyclovir, IVIG	Alive
	Lu [17]	14 M	ALL	hepatitis, DIC, Myocarditis	Acyclovir, WIG	Alive
Non-Solid organ transplantation associated	Maggi [18]	49 M	Healthy	Hepatitis	hepatectomy	Dead
	Babv-Defaux [19]	28 M	Healthy	Hepatitis. Pneumonia, Rhabdomyolysis DIC Encephalopathy	Acyclovir	Alive
	Plesek [20]	26 F	spinal demyelination syndrome	Hepatitis, DIC	Acyclovir	Dead
	Saitoh [21]	47 M	Multiple myeloma	Hepatitis, DIC, encephalopathy	Transfusion	Dead
	Wiegering [22]	4 M	Healthy	Puhnonary, Hepatitis, DIC	Acyclovir	Dead
	Roque-Afons [23]	63 F	Asthma and sinusitis	Fuluminant hepatic failure, DIC	Acyclovir, IVIG Liver transplantation,	Alive

*M* male, *F* female, *SLE* Systemic Lupus Erythematosus, *ALL* A cute Lympocytic Leukemia, *LDLT* Living donor liver transplantation, *DIC* disseminated intravascular coagulation, *IVIG* intravenous immunoglobulin

**Fig. 3:**
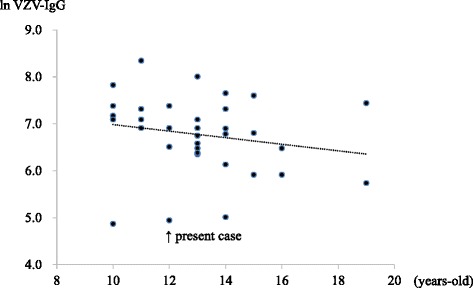
VZV-IgG values at LDLT in 36 patients received LDLTs at 10–20 years old in our institute. The values are shown as ln VZV-IgG titers. Three patients, including the patient in the present case, exhibited relatively low antibody titers. There existed one patient who didn’t have VZV-IgG and not be shown in the figure

We have previously reported our strategies for preventing VZV infection after pediatric LDLT, which was the largest study regarding VZV infection after pediatric LDLT [8]. Interestingly, Yamada et al. have also reported a case of severe disseminated VZV infection that occurred in an immunocompromised host, although that patient exhibited positive immunity [24]. However, we still believe that preoperative vaccination and positive immunity after the transplant are the critical considerations for managing patients after LDLT. We have performed 266 LDLTs between May 2001 and March 2015, including 36 (14.3 %) cases in which the patients were between 10-20-years-old at the LDLT; their VZV-IgG values at the LDLT are shown in Fig. 3. Among these cases we found that three patients (including this present case) exhibited relatively low VZV-IgG values and another one patient did not have VZV-IgG at the LDLT. Therefore, we currently believe that an additional vaccination may be added for similar patients before their LDLT, although it is difficult to select an appropriate VZV-IgG cut-off value for the additional vaccination. Besides, we are considering about performing re-vaccination for patients who don’t have positive immunity at 2 years after their LDLT.

In the present case, the patient had received an episode of steroid pulse therapy treatment and was receiving three immunosuppressants (FK, MP, and MMF). Although it is unknown whether the present case was primary infection or re-activation of the vaccine strain, it is possible that re-activation of the vaccine strain might have led to severe disseminated VZV infection [25–27]. Patients who are receiving a potent immunosuppressant regimen have a higher risk of latent infection or vaccine re-activation, and may develop severe illness if VZV infection occurs, regardless of whether it is primary infection or re-activation. Therefore, an early diagnosis and treatment are essential to rescue patients with disseminated VZV infection and MOF. In addition, when VZV infection is suspected in solid organ transplant recipients, it is critical to consider their vaccination history, anti-VZV titers, and presence of VZV-DNA via PCR, as this information can help facilitate an early and accurate diagnosis. If a diagnosis of disseminated VZV infection is made, appropriate early treatment is dictated by the patients’condition, and may include hospitalization, intravenous acyclovir, intravenous immunoglobulin, and the withdrawal of immunosuppressants.

The primary treatment for disseminated VZV infection with MOF should address the organ failure and involve antiviral therapy, including intravenous acyclovir. In general, the antiviral therapy should be continued until all skin rashes are covered with scabs, and the organ symptoms have disappeared. Interestingly, Kronenberg et al. have reported four cases of disseminated VZV infection after solid organ transplantation in which the VZV-DNA levels were monitored via PCR [28]. Among those cases, one patient exhibited an increase in VZV-DNA that was accompanied by recurrent gastritis. In our case, we monitored the VZV-DNA levels via PCR, and they remained high (8.5 × 10^3^copies/mL), even after all skin rashes and organ symptoms had completely resolved. Therefore, we added prophylactic oral acyclovir and continued monitoring until a decrease in the patient’s VZV-DNA was observed without any symptoms. This course appears to have been effective, as the patient has not experienced a recurrence. Similarly, monitoring for Epstein Barr virus DNA via PCR is and established method for prophylaxis and management of those infections, which are also common after liver transplantation [29, 30]. However, VZV-DNA monitoring via PCR is not an established method, although it appears to have the potential to be an effective biomarker for early diagnosis of VZV infection. In addition, this monitoring can evaluate the efficacy of treatment in cases of organ transplant recipients with severe disseminated VZV infection, especially in patients who have clinical symptoms of MOF.

In conclusion, although disseminated VZV infection with MOF after pediatric LDLT is a life-threatening disease, it can be cured via an early diagnosis and intensive treatment. In these cases, PCR monitoring of VZV-DNA is helpful to quickly reach an accurate diagnosis and evaluate the efficacy of the subsequent treatment.

## Consent

Written informed consent was obtained from the patient and her parents for publication of this case report and the accompanying images. A copy of the written consent is available for review by the Editor-in-Chief.

### Ethics

This study was approved by Ethics Committee of Jichi Medical University (rA15-042).

## Abbreviations

VZV: Varicella zoster virus
LDLT: Living donor liver transplantation
MOF: Multiple organ failure
FK: Tacrolimus
MP: Methylprednisolone
MMF: Mycophenolate mofetil
CMV: Cytomegalovirus
DIC: Disseminated intravascular coagulation
PCR: Polymerase chain reaction
IgG: Immunoglobulin G
